# Supplementary material for: Evaluation of wound healing and anti-inflammatory activity of hydro-alcoholic extract and solvent fractions of the leaves of Clerodendrum myricoides (Lamiaceae) in mice
Source: PLoS One. 2024 Jul 10;19(7):e0306766. doi: 10.1371/journal.pone.0306766 (PMC11236120; doi:10.1371/journal.pone.0306766)
Supplement: S2 File — (DOCX) [file pone.0306766.s002.docx]

Author checklist

| Item | Response | remark |
| --- | --- | --- |
| Study design | - Experimental study design were implemented - For the excision and burn wound models, mice were grouped in to four. - Group I was vehicle control group, - Group II and III were treatment groups and - Group IV was positive control group. - For the incision wound model, in addition to these four groups there are additional groups, group V which was Left untreated (untreated negative control). - For solvent fraction mice were grouped in to eight - Group I was vehicle control group, - Groups II and III were aqueous fraction treatment - Groups IV and V were ethyl acetate fraction treatment - Groups VI and VII were n-hexane fraction treatment - Group VIII was positive control group. - For anti-inflammatory activity, animals were assigned into five groups - Groups I : vehicle control group(2% Tween 80), - Group II: positive control (indomethacin 10mg/kg) - Group III : 100 mg/kg test extract , - Group IV : 200 mg/kg test extract - Group V : 400 mg/kg test extract - Six mice in each group were assigned - The result were compared between the groups |  |
| Sample size | - Experimental unit for each groups: six mice per group - Total number of mice per each experiment - Excision wound models: 24 mince - Burn wound models : 24 mice - Incision wound models : 30 mice - For solvent fraction : 48 mice - Anti-inflammatory : 30 mice - Acute dermal toxicity test : 5 mice - Total number of mice used: 161 mice - Sample size calculation is based on previous experimental study |  |
| Inclusion and exclusion criteria | - Healthy Swiss albino mice (either-sex) with 25-30 gm and 6-8 weeks of age were included in the study - Mice with Lacerated, wounded, and abnormal skin texture were excluded in the Study - All the mice included in the experiment study were included in the analysis - In each experimental group n=6 |  |
| Randomization | - Simple random method were used to allocate mice in groups - Each group of mice were placed in separate cage, the cage of the mice were labeled. - Each mice in the cage are given number( labeled in their tie using permanent marker) |  |
| Blinding | - The principal investigator(Alemante Tafese and wubayehu Kahaliw) aware groups allocation in conducting the experiment, analysis and outcome assessment) |  |
| Outcome measures | - Wound contraction, tensile strength, histopathology change, epithelialization period and paw edema reduction were the outcome variable in the study. |  |
| Statistical methods | - SPSS software (version 24.0) was used. - The result was statistically analyzed using a one-way ANOVA followed by a post-hoc Tukey test. - P <0.05 was considered statistically significant. |  |
| Experimental animals | - Healthy Swiss albino mice (either-sex) with 25-30 gm and 6-8 weeks of age were included in the study |  |
| Experimental procedures | - The experimental animals were handled and cared for according to the internationally accepted laboratory animal use, care, and welfare guidelines. - Control group were handled in the same manure like test group the only deference is types of medication applies, the control groups mice threated with simple ointment that are used to prepare the extract ointment. In the case of anti-inflammatory the control group receive vehicle with 2% tween 80 that used to dilute the extract. - All experimental groups include control groups are acclimatized in the laboratory room for a week before actual experiment begins. - **Acute dermal Toxicity;** - Dermal irritation evaluation was carried out according to OECD Guideline 404. - Since female mice are more sensitive to dermal toxicity than male mice, five healthy female Swiss albino mice with normal skin texture aged between 6 and 8 weeks were used. - **Excision Wound Model:** - Prior to wounds creation, mice were anesthetized with ketamine (80 mg/kg) and diazepam (5 mg/kg) by administering intraperitoneally. - The skin fur of the dorsolateral flank area 1–1.5 cm away from the vertebral column on either side and 3 cm away from the ear were shaved and disinfected with 70% alcohol. - The anticipated circular wound areas (300 mm^2^) were marked with a thin permanent marker and created a 2 mm-deep excised wound. The entire wound was left open to the external environment for 2 hours. After recovery, mice were returned to their cage and considered to be on day 0. The simple ointment (vehicle control), extract or solvent fractions, and standard drug (positive control) were applied topically once daily. - The wound healing capacities of the crude as well as a solvent fractions were evaluated by the percentage of wound contraction, period of epithelialization, and histological studies - **Incision Wound Model:** - Mice were anesthetized in the same manner as described for the excision wound model. - The dorsal fur of each mouse was shaved and decontaminated with 70% alcohol. Longitudinal paravertebral incision (3 cm long and 2 mm deep) was made with a sterile blade on either side at a distance of 1.5 cm from the dorsal midline. - Wounds were closed with interrupted sutures, 1 cm apart, with surgical sutures braided silk (no. 00) and left undressed. The wounding day was considered day 0.The respective treatments (simple ointment, crude extract, and nitrofurazone (0.2%)) were applied once daily topically to the animals of respective groups starting from 24 hours after wound creation (on the 1st day) until the 9th day, and one group was left untreated and served as an untreated negative control. - The stitch was extricated on the eighth day, and the skin-breaking strength of the wound was measured on the tenth day of post-wounding using a continuous water flow technique. - Breaking strength was compared among the groups. - **Burn Wound Model:** - The mice were anesthetized in the same manner as described for the excision wound model. - The dorsal fur of each mouse was shaved and decontaminated with 70% alcohol. The burn wound was created through hot molten beeswax at 80^o^c, which was poured into a cylinder with a 300 mm^2^ circular opening placed on the shaven back of the mice until the wax solidified. - The cylinder was removed after approximately 10 to 12 minutes of solidification, leaving the marked circular burn. The animal was placed in a separate cage. - Ointment was applied over the wound area with the respective groups as described in the grouping and dosing section every day, starting from day one until the day of the scab falling off the positive control. The progress of healing was examined every 2 days by measuring the percentage of wound contraction and epithelialization - **Anti-inflammatory Activity:** - As a model for acute inflammation, carrageenan-induced paw edema was employed. - We examined the anti-inflammatory activity of 80% hydrometanolic crude extract of the leaves of *C. myricoides* using Swiss albino mice of either sex. Before administering any medication, each mouse's right hind paw's basal volume was measured using a plethysmometer after an overnight fast with free access to water. Following the determination of the basal volume, the animals were grouped into five, each of which had six mice, with no significant mean volume difference. - The mice were given oral doses of 100 mg/kg, 200 mg/kg, and 400 mg/kg of the plant extracts before one hour of induced inflammation. The doses were selected based on the acute oral toxicity test from a previous study [22, 36], along with 10 mg/kg of indomethacin (positive control) and the vehicle (negative control, 2% Tween 80). To induce inflammation, 0.05 ml of a 1% carrageenan in 0.9% saline (w/v) solution was injected into the right hind paw's (sub-plantar) area of mice. - Using a water displacement plethysmometer (model: PLM 01 PLUS), the paw volume was measured at 0, 1, 2, 3, and 4 hours following the injection of the inflammatory stimuli (carrageenan). The percentage of edema inhibition in treated animals was calculated in comparison to the negative control group. The results obtained were compared among groups - The above all procedure are adopted from different publish study and different guidelines which are sited in the manuscript |  |
| Results | All values were presented as mean ± SEM |  |
|  |  |  |

| item | Response | remark |
| --- | --- | --- |
| Abstract | **Background:** Wounds significantly affect people's quality of life and the clinical and financial burden of healthcare systems around the world. Many of the current drugs used to treat wounds have problems such as; allergies and drug resistance. Hence, the exploration of new therapeutic agents from natural origin may avert this problem. *Clerodendrum myricoides* have long been used to treat wounds in Ethiopia. Despite this, nothing has so far been reported about the wound healing and anti-inflammatory activity of *C. myricoides*. This study aimed to evaluate the wound healing and anti-inflammatory activity of 80% methanol extract and solvent fractions of *C. myricoides* leaves in mice.  **Methods:** Leaves of *C. myricoides* were extracted using the maceration technique. The extract was formulated as 5% and 10% w/w ointments. The wound healing activity of the extract was evaluated using excision, incision, and burn wound models whereas the healing activities of solvent fractions were evaluated using the excision wound model. A carrageenan-induced paw edema model was used for the anti-inflammatory test.  **Results:** In the dermal toxicity test, 2000 mg/kg of 10% extract was found to be safe. In excision and burn wound models, treatment with 10% and 5% extract showed a significant (p<0.001) wound contraction. Solvent fractions of the extract significantly reduced wound contraction. A significant reduction in periods of epithelialization and favorable histopathology changes were shown by extract ointments. In incision wounds, 10% (p<0.001) and 5% (p<0.01) extracts significantly increase skin-breaking strength. After one hour of treatment, 400 mg/kg (p<0.001) and 200 mg/kg (p<0.05) showed significant reduction in paw edema.  **Conclusion:** Results of this study indicate that 80% methanol extract and the solvent fraction of the leaves of *C. myricoides* possess wound-healing and anti-inflammatory activity and support traditional claims. |  |
| Background | Wound is damage or disruption to the normal anatomical structure, function, and integrity of living tissue, and it arises from chemical, thermal, microbial, or physical damage to a tissue or be the result of a disease process. The healing of wounds is a complex and dynamic process of restoring the structure and function of damaged tissues .  Wounds, especially chronic wounds, have considerable humanistic and economic burdens. Chronic wounds represent a substantial financial and humanistic challenge for society. They not only diminish the quality of life for individuals grappling with them but also escalate healthcare expenses due to reduced productivity . According to current estimates, 1-2% of people in developed countries will encounter a chronic wound at some point in their lives. Despite wound care being a worldwide issue valued in the multibillion-dollar range, in the US alone, it impacts approximately 5.7 million individuals, accounting for roughly 2% of the population, and incurs an annual expenditure of US$20 billion Chronic wounds cost 1% to 2% of the yearly health care budget in European countries . In South Asian and sub-Saharan African countries, 1% to 2%of people have experienced a chronic wound at some point in their lives . The burden of wound is high in Ethiopia as reported in a study conducted in 2015 in Amhara Regional State Referral Hospitals, In this study, injury was found to 55.6% in visiting emergency departments .  Plant-based treatment is widely practiced in Ethiopia to facilitate the healing of these injuries. . In addition, medicinal plants are used for a number of conditions and are widely accepted across various cultures and socio-economic levels .  In Ethiopia, Clerodendrum myricoides, “Misirich” (Amharic) is used to treat wounds and burns (dried leaves are grounded, powdered and applied on infected part) , malaria , epilepsy and anthrax , conjunctiva, and trachoma . Previous reports indicated that C. myricoides showed in vitro antibacterial , and antioxidant activity . Studies in animals reported that, C. myricoides showed strong activities against P. berghei (antimalarial) , antidiarrheal , and diuretic effects . Though there is strong ethnobotanical evidence, there hasn't been any scientific research on the plant's ability to manage wounds. Therefore, the purpose of this study was to provide scientific proof supporting the plant's traditional use in wound healing. |  |
| Objectives | - General objective - To evaluate wound healing and anti-inflammatory activity of 80% hydromethanolic extract and solvent fractions of the leaves of Clerodendrum myricoides in mice - 2.2. Specific Objectives - To study acute dermal toxicity test in mice - To evaluate wound healing activity of 80% hydromethanolic crude extract and solvent fraction of the leaves of Clerodendrum myricoides on excision wound model in mice - To evaluate wound healing activity of 80% hydromethanolic crude extract of the leaves of Clerodendrum myricoides on incision wound model in mice - To evaluate wound healing activity of 80% hydromethanolic crude extract and solvent fraction of the leaves of Clerodendrum myricoides on burn wound model in mice - To evaluate anti-inflammatory effect of 80% hydromethanolic crude extract of the leaves of C. myricoides using carrageenan induced paw edema model in mice |  |
| Ethical statement | - An approved ethical clearance was requested and obtained from the Bioethics Committee of Department of Pharmacology, University of Gondar with a reference number Sop4/08/06/2015. |  |
| Housing and husbandry | - Mice are kept and breed in standard laboratory of Gondar university medical college. Which is well ventilated and having room thermometer and 12 hours on and off lighting system. |  |
| Animal care and monitoring | - To reduce pain and anxiety we have administered anesthesia ketamine (80 mg/kg) and diazepam (5 mg/kg) before actual experiment begin - After completing the experiment mice are discarded in human way (euthanasia) by using high dose of anesthesia. |  |
| Interpretation/scientific implications | - According to the result , our plant shows promising wound healing activity - As limitation we have use histopathology method to estimate collagen density due to an availability of hydroxyl proline in the laboratory |  |
| Generalisability/translation | - This result shows the effect of the plant in mice wound. It is difficult to generalized to human being since we have different anatomical and physiological difference. |  |
| Protocol registration | - Before actual experiment begin, basic research question, research objective and analysis plan were prepared and submitted in Gondar university pharmacy department and approved. |  |
| Data access | All relevant data are within the manuscript and it Supporting Information files. |  |
| Declaration of interests | - The authors have no conflict of interest to disclose - The project was not funded by any organization or institution |  |
