# Supplementary material for: Evaluation of wound healing and anti-inflammatory activity of hydro-alcoholic extract and solvent fractions of the leaves of Clerodendrum myricoides (Lamiaceae) in mice
Source: PLoS One. 2024 Jul 10;19(7):e0306766. doi: 10.1371/journal.pone.0306766 (PMC11236120; doi:10.1371/journal.pone.0306766)
Supplement: Attachment — Submitted filename: Review comments and suggestions for authors.pdf [file pone.0306766.s003.pdf]

The manuscript entitled “Evaluation of Wound Healing and Anti-Inflammatory Activity of Hydro-alcoholic Extract and Solvent Fractions of the Leaves of Clerodendrum myricoides (Lamiaceae) in Mice”, introduces new findings but requires significant revisions that undermine its originality.

1. The authors are advised to thoroughly review the manuscript to prevent any spelling or syntax mistakes.
2. On page 4, it is recommended that the authors include the latest data regarding the humanistic and economic impact.
3. In section 2.1 on page 5, the authors overlooked details regarding carrageenan and indomethacin.
4. On page 7, in section 3.2, the authors mistakenly referred to h-hexane instead of n-hexane.
5. The authors stated in section 3.4 on Page 9 that mice were categorized into four groups for excision and burn wound models, whereas for the incision model, the animals were separated into five groups. What are the factors that led to these differences in the grouping of animals?
6. In section 3.4, on Page 9, the authors overlooked the name of the drug used for the positive control group in the excision, incision, and burn models.
7. In section 3.5.1.1 on page 10, the authors should provide a clear and detailed explanation of the method used to measure the size of the wound.
8. On Page 10, in section 3.6.3, the authors addressed both excision and burn wounds. This section specifically pertains to excision wounds.
9. In the 3.7 section on page 11, the authors should include a detailed explanation of the water flow technique.
10. On page 12, the Anti-inflammatory Activity section lacks information about the specific plethysmometer technology used (water displacement or mercury) and the model of the plethysmometer.
11. On page 13, in section 4.2.1, the authors state that Figure 1 pertains to the excision wound model. However, on page 14, Figure 1 actually displays photos of burn wound healing.

12. Figure 1 displays photos of day 0 and day 20, however, the authors consider the day 16 readings as the ultimate measurements of the wound contractions.
13. . The researchers assess the size of the wounds every other day. It will enhance their study if they provide photographs of all time periods.
14. On page 18, the authors mentioned the highest percentage of epithelialization period (26.82%) reduction is produced by 0.2% nitrofurazone ointment while the lowest percentage of epithelialization period (26.82%) reduction is produced by 5% ethyl acetate fraction. This statement may be perplexing.
15. Label images (C and D) in Figure 3 on page 20.
16. Figure 4 on Page 21 displays photos of wounds that were taken on the same day they occurred. The suture was removed on the eighth day. It would be beneficial if they provide photographs of the eighth day following the removal of the sutures.
17. Include images for the burn wound model in the manuscript.
18. Specify the edema volume for 100 mg/kg extract in Table 9 on page 26.
19. Improve the clarity of photographs in Figure 1 and Figure 4.
20. Regarding the question of interest, it's noteworthy that while the manuscript discusses the wound healing and anti-inflammatory effects of Clerodendrum myricoides leaves, it lacks biochemical studies to assess antioxidant and anti-inflammatory markers.
