# Supplementary material for: Evaluation of wound healing and anti-inflammatory activity of hydro-alcoholic extract and solvent fractions of the leaves of Clerodendrum myricoides (Lamiaceae) in mice
Source: PLoS One. 2024 Jul 10;19(7):e0306766. doi: 10.1371/journal.pone.0306766 (PMC11236120; doi:10.1371/journal.pone.0306766)
Supplement: Attachment — Submitted filename: Response to Reviewer.docx [file pone.0306766.s004.docx]

**Response to reviewer**

**Title:** “Evaluation of Wound Healing and Anti-Inflammatory Activity of Hydro-alcoholic Extract and Solvent Fractions of the Leaves of Clerodendrum myricoides (Lamiaceae) in Mice”

**Manuscript submission number:** PONE-D-24-09941

**Journal:** PLOSE ONE

**Response to editor**

We are very thankful to you for handling the publication process of this manuscript. Modified portions or newly added texts in the revision process are marked-up with green color, whereas deletions are shown by red colored texts with Strikethrough line, ~~example.~~

**Response to reviewer**

I wanted to extend my heartfelt appreciation for your diligent review of my manuscript. Your expertise and insightful feedback have been invaluable in shaping the development of this work.

I have carefully reviewed the reviewer's comments and suggestions, and I am grateful for the thoroughness with which they were provided. Your constructive criticism has guided me in refining the manuscript to ensure its accuracy and clarity.

Attached is the revised version of the manuscript, which incorporates the changes, recommended by the reviewer. I trust that these revisions have addressed the concerns raised and have strengthened the overall quality of the manuscript.

**Question raised by reviewer (Q: Question, A: answer)**

**Q1:** The authors are advised to thoroughly review the manuscript to prevent any spelling or syntax mistakes.

**A1:** We appreciate the reviewer's feedback and have reviewed the manuscript to correct any spelling or syntax mistakes.

**Q2:** On page 4, it is recommended that the authors include the latest data regarding the humanistic and economic impact.

**A2:** Accepted, we include the latest data regarding the humanistic and economic impact to enhance the relevance and completeness of the manuscript.

**Q3:** In section 2.1 on page 5, the authors overlooked details regarding carrageenan and indomethacin.

**A3:** we acknowledge the oversight regarding carrageenan and indomethacin. We include the necessary information to provide a comprehensive understanding.

**Q4:** On page 7, in section 3.2, the authors mistakenly referred to h-hexane instead of n-hexane.

**A4:** Thanks, we correct the typing error from "h-hexane" to "n-hexane".

**Q5:** The authors stated in section 3.4 on Page 9 that mice were categorized into four groups for excision and burn wound models, whereas for the incision model, the animals were separated into five groups. What are the factors that led to these differences in the grouping of animals?

**A5:** The differences in grouping animals for the excision, burn, and incision models were based on the specific requirements and protocols tailored for each experimental procedure. In incision wound model there is additional group five which is left untreated (untreated negative control) group. This because, additional left untreated group are added for comparison purposes. This group essentially serves as a control, similar to the control group in the other models. Its purpose is to provide a baseline for the wound healing process in the absence of any treatment. By including this left untreated group, it can better evaluate the effectiveness of the treatments being tested by comparing them not only against each other but also against the natural progression of wound healing.

**Q6:** In section 3.4, on Page 9, the authors overlooked the name of the drug used for the positive control group in the excision, incision, and burn models.

**A6:** we apologize for the oversight regarding the name of the drug used for the positive control in each group. We are included in the revised manuscript.

**Q7:** In section 3.5.1.1 on page 10, the authors should provide a clear and detailed explanation of the method used to measure the size of the wound.

**A7:** Accepted, we provide a clear and detailed explanation of the method used to measure the size of the wound as suggested.

**Q8:** On Page 10, in section 3.6.3, the authors addressed both excision and burn wounds. This section specifically pertains to excision wounds.

**A8:** we refined to focus specifically on excision wounds as recommended.

**Q9:** In the 3.7 section on page 11, the authors should include a detailed explanation of the water flow technique.

**A9:** we included a detailed explanation of the water flow technique as requested.

**Q10:** On page 12, the Anti-inflammatory Activity section lacks information about the specific plethysmometer technology used (water displacement or mercury) and the model of the plethysmometer.

**A10:** we are used water displaced method and PLM 01 PLUS model of the plethysmometer in the Anti-inflammatory Activity section. It is included in the updated manuscript.

**Q11:** On page 13, in section 4.2.1, the authors state that Figure 1 pertains to the excision wound model. However, on page 14, Figure 1 actually displays photos of burn wound healing.

**A11:** thank you very much; we corrected the discrepancy between the description of Figure 1 and its content as pointed out by the reviewer.

**Q12**: Figure 1 displays photos of day 0 and day 20, however, the authors consider the day 16 readings as the ultimate measurements of the wound contractions.

**A12:** Figure 1 display photo of burn wound model and the reading day for burn wound is 20 day. Day 16 is for excision wound model. However, we clarified the measurement time points for wound contractions to accurately reflect the study methodology

**Q13:** The researchers assess the size of the wounds every other day. It will enhance their study if they provide photographs of all time periods.

**A13:** We provided photographs in time periods in the revised manuscript.

**Q14:** On page 18, the authors mentioned the highest percentage of epithelialization period (26.82%) reduction is produced by 0.2% nitrofurazone ointment while the lowest percentage of epithelialization period (26.82%) reduction is produced by 5% ethyl acetate fraction. This statement may be perplexing.

**A14:** Thanks, We revised the statement and corrected in the revised manuscript.

**Q15:** Label images (C and D) in Figure 3 on page 20.

**A15:** The images (C and D) in Figure 3 on page 20 are appropriately labeled as suggested.

**Q16**. Figure 4 on Page 21 displays photos of wounds that were taken on the same day they occurred. The suture was removed on the eighth day. It would be beneficial if they provide photographs of the eighth day following the removal of the sutures.

**A16.** Unfortunately, I didn't take a photo on the eighth day after suture removal.

**Q17.** Include images for the burn wound model in the manuscript.

**A17.** Accepted, Image for excision and burn wound model are updated in revised manuscript.

**Q18.** Specify the edema volume for 100 mg/kg extract in Table 9 on page 26.

**A18.** The edema volume for 100 mg/kg extract in Table 9 on page 26 are specified as requested

**Q19.** Improve the clarity of photographs in Figure 1 and Figure 4.

**A19.** We are improving the clarity of photographs and updated in revised manuscript.

**Q20.** Regarding the question of interest, it's noteworthy that while the manuscript discusses the wound healing and anti-inflammatory effects of Clerodendrum myricoides leaves, it lacks biochemical studies to assess antioxidant and anti-inflammatory markers.

**A20.** While the manuscript primarily focuses on wound healing and anti-inflammatory effects, we acknowledge the suggestion to include biochemical studies to assess antioxidant and anti-inflammatory markers. We will consider this for future research endeavors.

Thank you for the valuable feedback, and we are committed to addressing all concerns to enhance the quality and clarity of the manuscript.
